# Discrete element modeling of the effect of real-shape ballast angularity on sleeper lateral resistance

**DOI:** 10.1038/s41598-025-31965-4

**Published:** 2025-12-11

**Authors:** Jafar Chalabii, Richard Nagy, Majid Movahedi Rad

**Affiliations:** 1 Department of Structural and Geotechnical Engineering, Széchenyi István University, Egyetem tér 1, Győr, 9026 Hungary; 2 Department of Transport Infrastructure and Water Resources Engineering, Széchenyi István University, Győr, Hungary

**Keywords:** Ballast angularity, Discrete element method, Lateral resistance, 3D scanning, Real-Shape modeling, Engineering, Materials science

## Abstract

The mechanical behavior of railroad trackbeds, especially their lateral resistance under dynamic train loads, is significantly influenced by ballast angularity. Using simulations using the Discrete Element Method and realistic particle geometries acquired through 3D scanning, this study examines the function of ballast particle angularity. An Artec Space Spider was used to scan and import five ballast samples into PFC3D, each of which had a unique size distribution and angularity index. To simulate a Single Tie Push Test, a B70 concrete sleeper, which is frequently found in European tracks, was modelled and put through lateral loading. Results for the standard No. 24 ballast gradation were compared with experimental data to validate the simulation framework, and the results indicated a high degree of agreement in the lateral force–displacement behavior. By examining changes in the particle size distribution, ballast degradation was measured, and the resulting Ballast Breakage Index and Breakage Ratio were calculated. Using accepted techniques, lateral resistance was calculated as the area under the displacement curve at 3.5 mm. According to the results, samples with more angular particles had lower degradation and higher lateral resistance. The importance of angularity in stabilising ballast layers under lateral loads was validated by regression analysis. These results offer guidance for better ballast selection and maintenance practices in the field of railway engineering.

## Introduction

The lateral stability of ballasted railway tracks is highly dependent on the resistance provided by concrete sleepers, which are essential for maintaining alignment and overall track integrity. A key aspect of this resistance is the mechanical interlocking between ballast particles and the sleeper surface. Ballast characteristics such as particle shape, size, angularity, and inter-granular friction-crucially influence load transfer mechanisms and shear strength within the ballast layer. While research has extensively examined ballast degradation, particle grading, and fouling, there has been relatively little focus on how particle angularity alone affects lateral resistance. Angularity, which reflects the sharpness and irregularity of particle edges, directly impacts contact behavior and shear strength. Several researchers have investigated how particle angularity and geometry influence ballast shear strength and interlocking. Krengel et al.^1^ employed DEM simulations to explore interactions between particle shape and inter-granular friction. They observed that spherical particles exhibit a steady increase in shear resistance as friction rises; however, angular particles show a peak in resistance followed by a decline, attributed to limitations in particle rotation and altered contact dynamics. These findings emphasize the need for realistic three-dimensional modelling of angular ballast rather than idealised spheres.

While particle angularity governs interlocking and frictional behaviour, ballast breakage and abrasion also play a critical role in altering these contact conditions over time. Therefore, the following group of studies focuses on how degradation and breakage processes influence ballast performance. Ballast degradation through breakage or abrasion also significantly influences sleeper resistance and long-term track performance. Hu et al.^2^ developed an energy-centric model to characterize the crushing behavior of individual ballast grains, arguing that traditional stress–strain descriptions fall short when particles undergo multi-stage breakage accompanied by significant energy dissipation. Their findings emphasize that a particle-level energy framework offers a clearer sensitivity to failure processes than classic strength curves. Furthermore, recent studies have combined numerical and experimental techniques to gain a deeper understanding of the mechanical response of both new and deteriorated ballast. Using a combination of discrete element simulations and laboratory testing, Binaree et al.^3^ evaluated the macro- and micro-mechanical characteristics of ballast. Their results established a connection between microscopic morphological deterioration and macroscopic mechanical performance, showing that surface wear and angularity reduction significantly reduce shear strength and stiffness. Ballast degradation mechanisms like abrasion and edge rounding have a direct impact on interlocking behavior and lateral resistance, as this study shows. This emphasizes the importance of taking degradation effects into account when assessing ballast stability. Their findings further underscore the importance of capturing both micro- and macro-mechanical changes in ballast morphology, a concept directly aligned with this study’s DEM-based evaluation of angularity effects. In a related investigation, Rohrman, Kashani, and Ho^4^ examined ballast samples subjected to natural abrasion. Although the peak strength of these abraded particles remained comparable to fresh ballast, the worn samples displayed notably increased volumetric strain and settlement during load application signaling a potential decline in lateral stability for the track structure. Further numerical and experimental studies have confirmed the importance of crushing and abrasion.Benmebarek and Movahedi Rad^5^ employed DEM to simulate breakage behavior in cemented granular assemblies under pile penetration. Their work highlights how evolving microstructure specifically inter-particle contact evolution and fracture events can significantly alter load-bearing capacity and deformation response in engineered soil systems. Their findings showed that localized crushing near the pile tip significantly reduces penetration and shaft resistance, confirming the critical role of particle crushing in load transfer mechanisms. Earlier work by the same authors^6^ focused on the influence of contact conditions on particle fracture mechanisms, showing that coordination number, contact location, and contact area significantly affect particle strength-especially under complex loading conditions modeled with the bonded particle method. With advancements in numerical modeling, researchers have employed DEM to analyze ballast behavior under various conditions. Li et al.^7^ introduced a Bonded Particle Model (BPM) for irregular ballast particles, using Voronoi cutting and Minkowski Sum theory to simulate fracture behaviors and lateral resistance in ballast beds. Their results demonstrated that a mixed fracture mode combining normal and tangential stresses effectively replicates ballast deformation and breakage mechanisms. Complementary research (e.g., Benmebarek et al.^8^; Cui et al.^9^) further linked particle breakage to reductions in peak shear stress and to higher settlement under cyclic and dynamic loading.

In the present study, however, the physical process of particle breakage was not modeled directly within the DEM framework. Instead, different particle-size distributions (PSDs) were adopted to represent the effects of ballast degradation indirectly, under the assumption that breakage and abrasion had already occurred before testing. This simplified approach follows prior work^10^, where variations in PSD were used to model the influence of degradation on track stability.

The impact of ballast fouling and degradation on lateral resistance has also been widely studied. Koohmishi et al.^11^ applied infrared thermography to assess ballast fouling, revealing that fouled ballast exhibits distinct thermal signatures compared to clean ballast, paving the way for non-destructive evaluation techniques in railway maintenance. Liang et al.^12^ extended prior studies by investigating how heat transfer mechanisms operate within fouled ballast layers. Their results showed that variables such as solar radiation and air temperature had a noticeable impact on the temperature contrast between clean and contaminated ballast insights that could improve on-site detection techniques for track fouling. More recently, data-driven and sustainability-oriented research has addressed degradation and recycling aspects. Gong and Qian^13^, proposed an RGB imaging approach to estimate the Fouling Index (FI), offering a fast and cost-effective alternative to the conventional sieve-based analysis for assessing track performance. Sustainability in railway infrastructure has become an increasingly prominent focus in recent years. To support recycling efforts, Kwunjai et al.^14^ utilized high-resolution 3D scanning combined with machine learning to differentiate between fresh and degraded ballast. Their approach tracked small shape changes sphericity and convexity and linked them to how well particles lock together, supporting ballast reuse and reducing environmental impacts. Koohmishi and Guo^15^ demonstrated that mixing crumb rubber with ballast improves wear resistance and extends service life.

Collectively, previous research indicates that ballast integrity is governed by both particle geometry and degradation state. However, this study does not attempt to model breakage directly; degradation is represented indirectly through predefined PSD variations, consistent with the literature. Building upon this micro-scale understanding, recent studies have broadened toward system-level analyses that link material properties to overall track stability. Beyond individual particle behavior, a number of studies have explored how track-level features affect overall stability. Chi et al.^16^ tested under-sleeper pads (USPs) on ballasted tracks and found 8.5% less longitudinal resistance and 8.7% better vibration damping, gains that can prolong track assets. Zhang et al.^17^ studied polyurethane-solidified ballast (PSB) using laboratory and DEM simulations, finding that higher polyurethane content greatly increases compressive strength, making PSB suitable for high-load environments. Further DEM analyses by Chalabii et al.^10^ explored particle size distribution, sleeper geometry, and contact conditions, demonstrating how gradation and sleeper dimensions govern lateral resistance. Benmebarek and Rad^18^ modeled the effect of rolling resistance coefficients on coarse sand, showing that shear stress and volumetric change depend strongly on inter-particle rolling. Finally, Chalabii and Movahedi Rad^19^ investigated gradation effects and reported that finer distributions consistently reduce shear stress, underlining the importance of proper PSD design.

When realistic particle geometries are considered, however, few studies have isolated the effect of angularity alone on sleeper lateral resistance. Despite significant advancements in our understanding of ballast breakage and system stability, little is known about the precise impact of ballast angularity on sleeper lateral resistance, especially when realistic particle geometries and multiple gradations are taken into account. Using high-fidelity DEM simulations with 3D-scanned particle shapes, this work fills that gap. The study offers useful advice for resilient track design, ballast selection, and maintenance by establishing a correlation between angularity indices and simulated lateral resistance. From a practical standpoint, maintaining angular ballast during track maintenance can reduce lateral displacement of sleepers and extend tamping intervals. The validated correlation between Angularity Number and lateral resistance offers a quantitative basis for specifying ballast quality in design and renewal guidelines.

## Determine particle size distribution

**Fig. 1:**
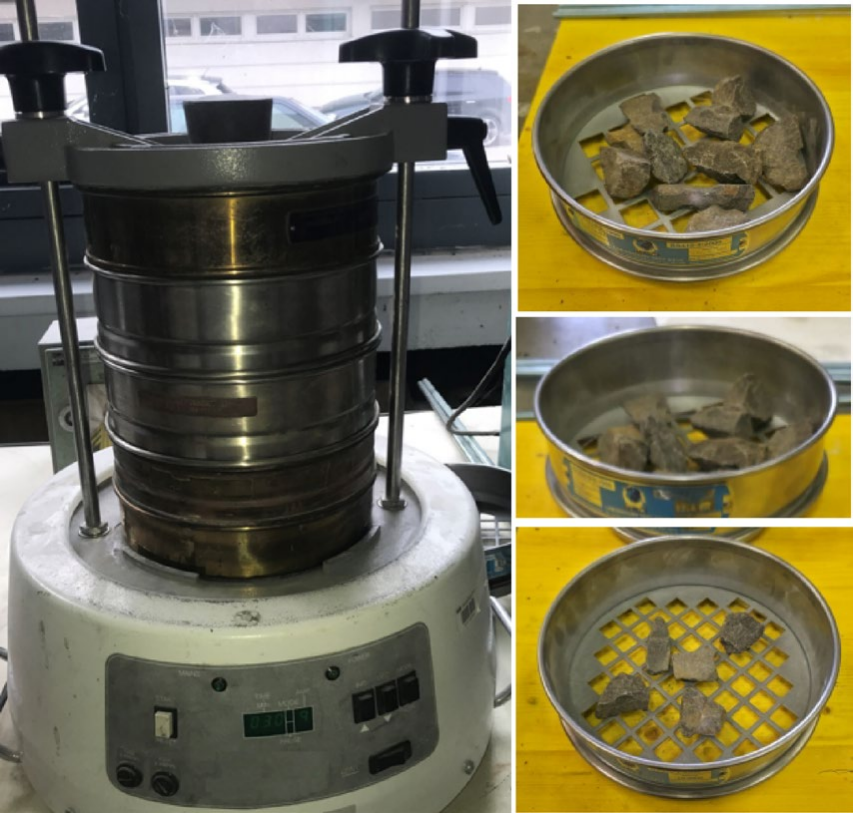
The digital electromagnetic sieve shaker.

To determine PSD of the ballast samples, a mechanical sieve analysis was performed using an Endecotts OCTAGON Digital electromagnetic sieve shaker, as shown in Fig. 1.

**Fig. 2:**
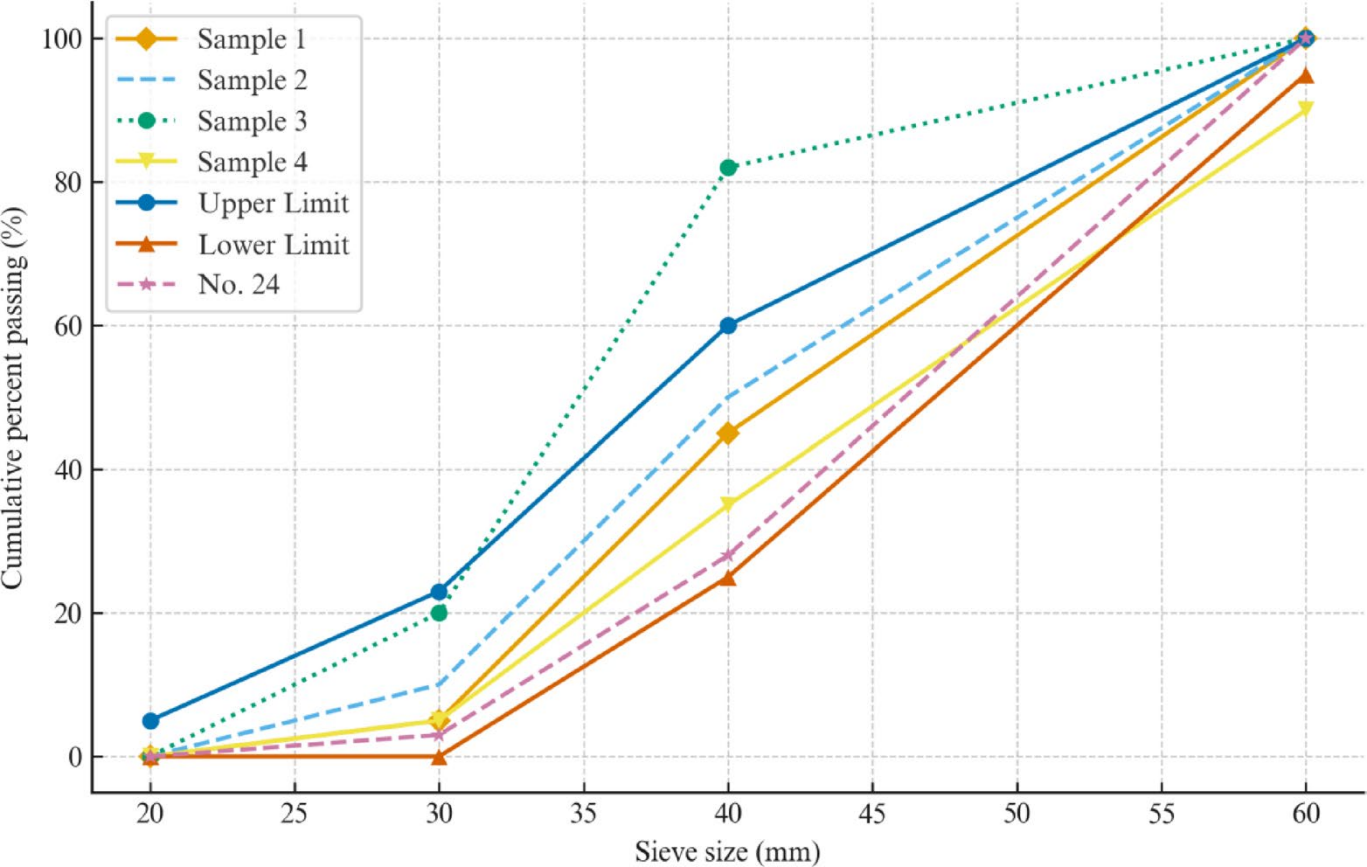
Particle size distribution of samples.

A mechanical sieve shaker was employed to perform particle size analysis, utilizing a stack of sieves arranged from largest to smallest mesh openings, consistent with ASTM C136 guidelines^21^. Each ballast sample was placed atop the sieve assembly and subjected to controlled vibration to ensure proper stratification based on particle size. The mass retained on each sieve was recorded, and from this, the percentage passing for each size range was determined. The resulting gradation data, visually represented through the curves in Fig. 2, served as the basis for classifying the samples and establishing boundary conditions for the DEM modelling scenarios.

As illustrated in Fig. 2, the five samples display noticeable differences in particle size distribution (PSD). Samples 1 and 2 contain a higher proportion of coarse particles (retained above 31.5 mm), while Samples 3 and 4 exhibit finer gradations with greater mass fractions below 19 mm. Sample 5 follows the standard No. 24 grading, providing a balanced curve that lies between the upper and lower specification limits. The upper and lower gradation bands in Fig. 2 correspond to the AREMA^22^ specifications for ballast No. 24. These variations enable assessment of how gradation influences ballast packing and resulting lateral resistance.

The measured gradation curves of the five ballast samples were used to create the particle assemblies in the DEM framework. These gradation curves were utilized as preset input conditions to represent various stages of the ballast condition because the degradation process was not explicitly modeled. Particle breakage was not specifically simulated in this study’s DEM analysis. Instead, under the presumption that breakage and abrasion had already taken place before testing, various particle size distributions (PSDs) were used to represent the effects of ballast degradation in an indirect manner. This simplification is in line with earlier research ch^10^ which used PSD variations to capture the impact of breakage on the lateral stability of ballasted tracks.

## Determine angularity number

The sharpness of ballast stones affects how well they grip one another, which in turn shapes the track’s ability to resist shear and side-to-side motion. Rough, angular grains with jagged edges lock together tightly, raising both the load they can bear and their resistance to sliding. Smooth, rounded pieces, by contrast, tend to slide past each other easily, weakening shear strength and making the track more prone to sideways drift under repeated train passes^23,24^. To study this effect, the team used the Angularity Number (AN) test to measure the shape of ballast across several gradations. The goal was to see how these angularity differences altered the sideways load-carrying behavior of concrete sleepers, and for that a Discrete Element Method (DEM) model provided the virtual test bed.

To represent real field conditions, five ballast batches were prepared according to ASTM C136 so that their particle size distributions matched specification limits. Each batch was sieved through the full set of screens, from 50 mm down to 4.75 mm, allowing a clear profile of coarse and fine components. Before the angularity tests, the stones were dried in an oven at 110 5 C until they reached constant weight. The mass held on every sieve was then recorded to build accurate PSD curves for subsequent numerical modelling.

**Fig. 3:**
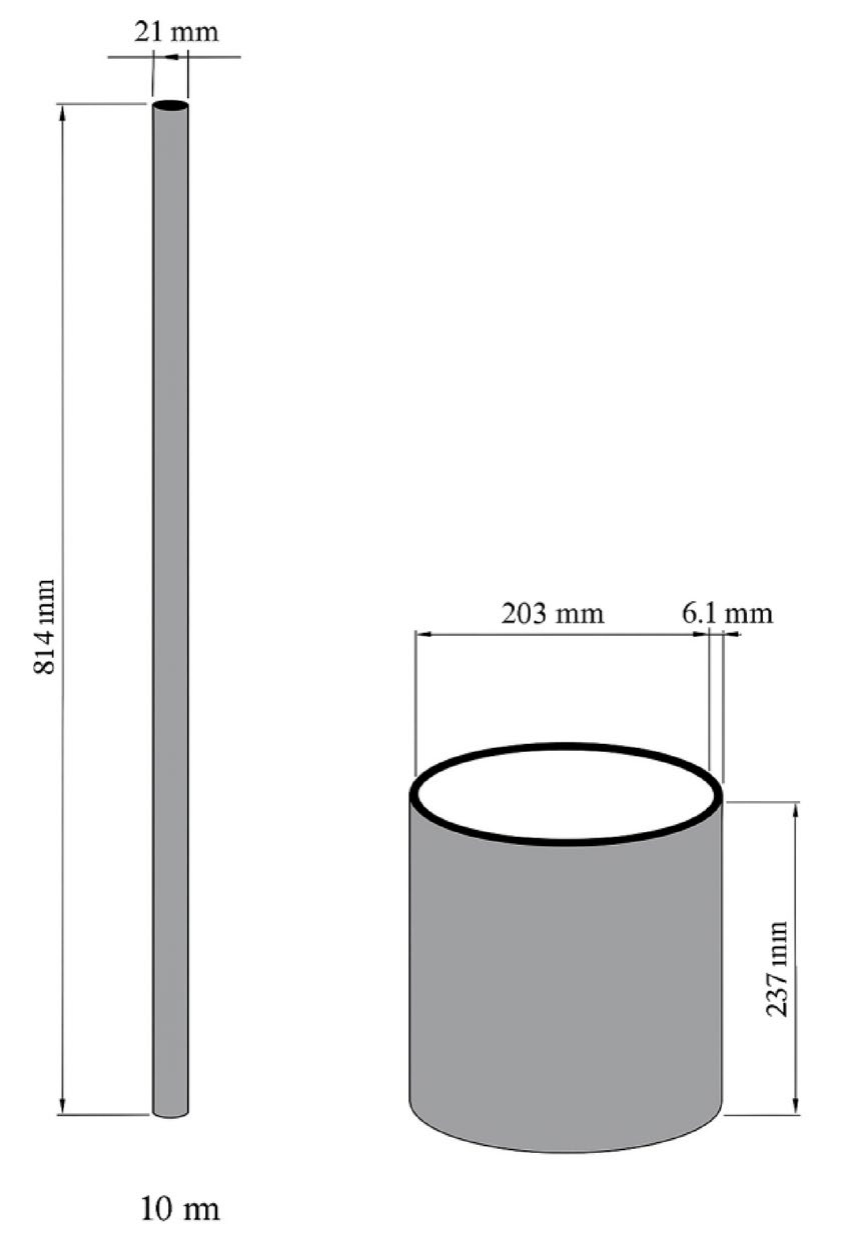
Dimension shape of the mold and the rod used in the laboratory.

**Fig. 4:**
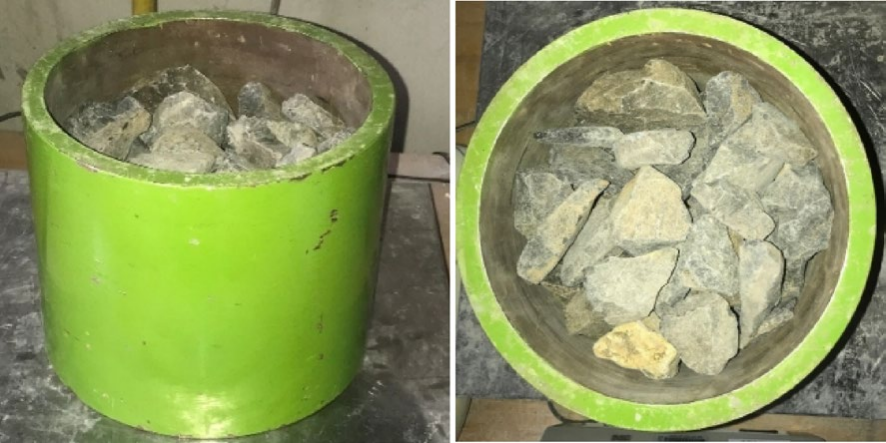
The mold with ballast in the laboratory.

Figure 2 presents the proportional mass distribution of each sample. The AN of the ballast materials was determined following the ASTM D3398-00^25^. This method measures the uncompacted void content of coarse aggregates, providing an index that reflects particle angularity and surface texture. The volume of the cylindrical mold was calibrated using the water displacement method. The mold held 2.574 kg of water at room temperature, giving a calibrated volume of 2.574 L. The aggregate was placed into the mold in three equal layers, each tamped 25 times using a steel rod equipped with a loose-fitting sleeve to ensure a 50 mm drop height, as specified in ASTM D3398^25^. The schematic configuration of the mold and tamping rod utilized for the angularity test is illustrated in Fig. 3. Additionally, the actual mold setup filled with ballast material, as used in the laboratory testing, is depicted in Fig. 4.

The filled mold was weighed, and the net sample mass was calculated by subtracting the known mold mass. Angularity Number was computed based on uncompacted void content using in Eq. (1).

1 $$\:{V}_{c}\left(\%\right)=\frac{{V}_{m}-\frac{{M}_{b}}{{G}_{b}{\beta\:}_{w}}}{{V}_{m}}$$

In this Equation, $\:{V}_{m}\:$represents the volume of the mold in $\:{cm}^{3}$, $\:{M}_{b}$ is the mass of the ballast sample in grams, $\:{G}_{b}$ denotes the specific gravity of the aggregate, which is assumed to be 2.70 based on typical values for crushed stone aggregates as specified in ASTM C127, and $\:{\beta\:}_{w}$​ is the density of water, taken as 1$\:\:g/{cm}^{3}$ according to standard laboratory conditions outlined in ASTM D854. In addition, $\:{V}_{c}\left(\%\right)$ is the percentage of uncompacted void content.

AN was then derived from the calculated uncompacted void content. It is determined using the following equation Eq. (2):

2 $$\:AN=\left(\frac{100-{V}_{c}\left(\%\right)}{0.6}\right)$$

This relationship is based on the empirical framework outlined in ASTM C1252^21^ for fine aggregates and extended to coarse aggregates through the methodology described in ASTM D3398^25^. Higher AN values correspond to particles with more angular shapes and rougher textures, indicating a greater degree of interlock potential. Experimental values for the five samples showed variation in AN, which reflects differences in angularity caused by gradation and material origin. These measured values were later used to correlate angularity with lateral resistance behavior observed in the DEM simulations. Table 1 presents the ballast mass, uncompacted void content, and the corresponding AN for five tested samples. The results show slight variations in ballast mass and void content, with AN values ranging between 101.85 and 102.60, indicating consistent particle angularity across samples.

**Table 1:** Related parameter to $\:AN$.

Sample	Ballast’s mass (g)	The uncompacted void content (%)	Angularity Number
	$\:{M}_{b}$	$\:{V}_{c}$	$\:AN$
1	$\:G$	$\:Pa$	–
2	$\:\nu\:$	–	–
3	$\:{\gamma\:}_{b}$	$\:Kg/{m}^{3}$	$\:2600$
4	$\:{\gamma\:}_{s}$	$\:Kg/{m}^{3}$	$\:2500$
5	$\:FI$	–	$\:0.9$

## 3D scanning

**Fig. 5:**
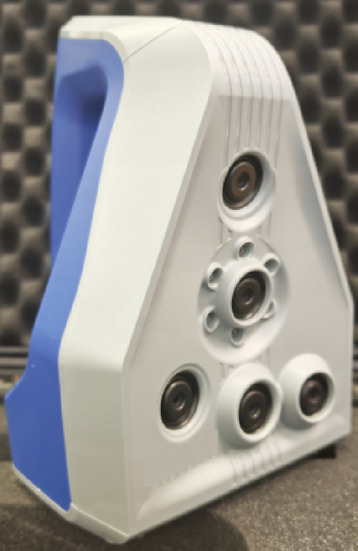
Artec Space Spider 3D scanner.

To accurately replicate the physical morphology of ballast aggregates, a high-precision Artec Space Spider 3D scanner was utilized, as shown in Fig. 5. This device uses blue LED light and operates without requiring surface markers, allowing for real-time scanning at 7.5 frames per second. It offers a 3D resolution of up to 0.1 mm and a point accuracy of 0.05 mm, making it highly suitable for detailed particle shape analysis^26^.

**Fig. 6:**
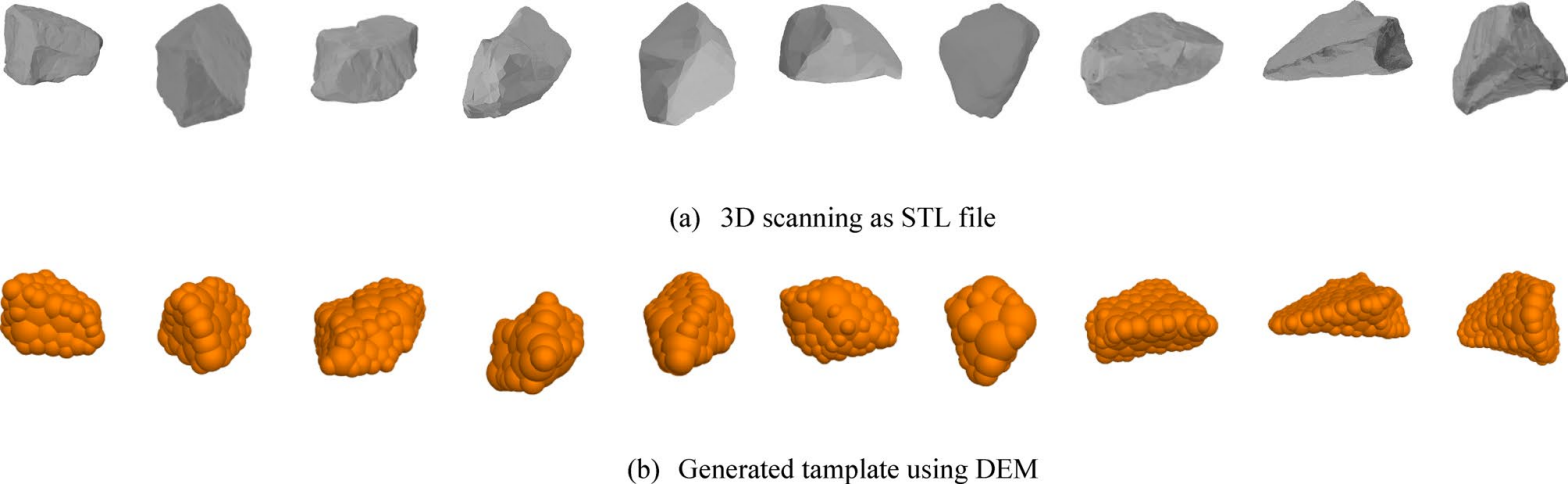
Comparison between scanned particle meshes and the corresponding clump templates used in DEM. The top row (a) shows STL files generated from 3D scanning, while the bottom row (b) displays their equivalent clump assemblies within PFC3D.

Each ballast sample, previously sorted by PSD, was scanned individually. In total, ten commonly occurring ballast particle shapes were selected and scanned to represent a realistic variety of geometries. The selected particles covered the full PSD range, including coarse (40–60 mm), medium (20–40 mm), and fine (below 20 mm) fractions, ensuring that shape variation was adequately represented across particle sizes. Selection was based on visual inspection and frequency of occurrence within each gradation class. The scanner required only a brief 3-minute warm-up to reach its operational temperature of 36.6 °C, ensuring consistent accuracy. The resulting 3D meshes were processed using Artec Studio 18 software and exported in STL format for implementation into the DEM simulations. Figure 6 displays the scanned particle meshes next to the clump templates made for DEM, clearly showing how real shapes turn into a model the computer can use. This indicates the one-to-one correspondence between real particle geometry and the simplified numerical representation. By following this step, the team slipped authentic particle forms straight into the code, so contacts in the virtual world now feel much closer to what happens in the lab.

Ten representative particles were selected to span the coarse, medium, and fine fractions of the PSD, ensuring that the DEM clump templates reflected the full size range and shape diversity of the ballast samples.

## DEM simulation

The Discrete Element Method (DEM) is a numerical technique developed to simulate the mechanical behavior of discontinuous materials, such as rocks and granular soils. Originally introduced by Cundall for analyzing rock mechanics problems^27^, DEM used deformable polygonal blocks to model the movement and interaction of individual elements. As the method evolved, its use expanded into the fields of soil mechanics and geotechnical engineering, establishing DEM as a foundational tool in the analysis of particulate systems. Building on its early development, specialized software like UDEC (Universal Distinct Element Code) and 3DEC (Three-Dimensional Distinct Element Code) were created to efficiently handle both 2D and 3D simulations involving blocky structures. Later, the Particle Flow Code (PFC) was introduced to extend DEM capabilities by enabling fast, accurate simulation of rigid particle interactions. In PFC, particles are idealized as rigid discs in two dimensions (PFC2D) and as rigid spheres in three dimensions (PFC3D), allowing for significant gains in computational efficiency and stability^28^.

In this research, Discrete Element Method (DEM) simulations were conducted using PFC3D version 7.0 to model the interaction between ballast and a B70-type concrete sleeper subjected to lateral loading. The simulation setup including geometry, boundary conditions, and contact models was adapted from the works of Khatibi et al. and Chalabii et al.^10,29^, with necessary modifications to suit the objectives of this study. A comprehensive overview of the material properties assigned to both the sleeper and ballast, along with the simulation methodology, is presented in the subsequent sections.

### Contact model

**Fig. 7:**
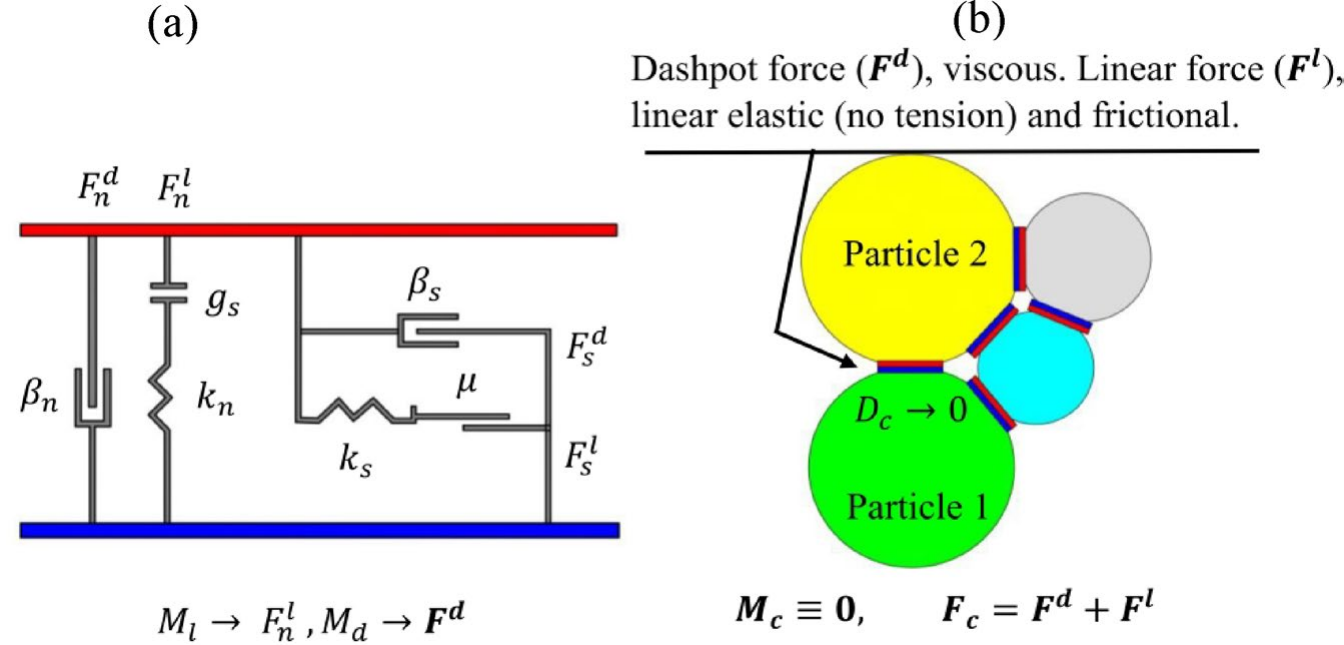
Mechanical behaviour and force interaction in the linear contact model: (a) schematic showing vertical and horizontal force components acting within the model, and (b) illustration of linear contact mechanics between some particles, adapted from the referenced study^30^.

In this work, we adopted a basic linear contact approach to capture how particles touch and slide in the DEM code. The method computes contact forces from simple linear links between overlap distance and chosen stiffness values. Normal and shear forces are determined independently, with shear forces capped by a Coulomb friction limit. The model is simple, computationally efficient, and well-suited for simulating granular materials^27^. Figure 7 illustrates the mechanical behavior and force interactions within the linear contact model: Fig. 7a presents a schematic showing the vertical and horizontal force components, while Fig. 7b visualizes the linear contact mechanics between particles. This concept was adapted from a previous study^30^ and provides the theoretical basis for the particle interactions used in this simulation. As illustrated in Fig. 7a, the linear contact model incorporates both normal and tangential force components, denoted as $\:{F}_{n}^{l}$ and $\:{F}_{s}^{l}$​, respectively. Complementary to these are the damping forces $\:{F}_{n}^{d}$​ in the normal direction and $\:{F}_{s}^{d}$​ in the shear direction. Critical model parameters include the damping ratios in both normal $\:{\beta\:}_{n}$ and tangential $\:{\beta\:}_{s}$ directions, the shear modulus $\:{g}_{s}$, and the coefficient of friction $\:\mu\:$. The symbols $\:{M}_{l}$​, $\:{M}_{d}$​, $\:{F}_{c}$​, and $\:M$ correspond to the linear force computation mode, damping mechanism, contact force vector, and the resulting contact moment.

In modeling particle interactions using a linear elastic contact law, several mathematical representations exist. In this study, the formulation proposed by Mindlin and Deresiewicz^31^ has been selected due to its established effectiveness in DEM-based analyses^32–36^. The normal contact stiffness $\:{K}_{n}$​ was calculated using Eq. (3), which depends on the shear modulus $\:{G}_{s}$​, effective radius $\:{R}_{e}$, normal overlap $\:{\delta\:}_{n}$​, and Poisson’s ratio $\:\nu\:$^34^.

3 $$\:{K}_{n}=\frac{{2G}_{s}{\left(2{R}_{e}{\delta\:}_{n}\right)}^{\frac{1}{2}}}{3(1-\nu\:)}$$

Similarly, the shear contact stiffness $\:{K}_{s}$​ was determined using Eq. (4), which incorporates the shear modulus, normal contact force $\:{F}_{n}$​, and other material parameters:

4 $$\:{K}_{s}=\frac{2}{2-\nu\:}{\left(3{{G}_{s}}^{2}{F}_{n}{R}_{e}\right(1-\nu\:\left)\right)}^{\frac{1}{3}}$$

In contact mechanics involving two spherical particles particle A with radius $\:{R}_{A}$​ and particle B with radius $\:{R}_{B}$​ the effective particle radius, represented as $\:{R}_{e}$​, is calculated using the harmonic mean of the individual radii. This relationship is expressed in Eq. (5):

5 $$\:\frac{1}{{R}_{e}}=\frac{1}{{2R}_{A}}+\frac{1}{2{R}_{B}}$$

This formulation ensures that contact forces are appropriately scaled relative to particle size during simulation. The normal contact force, denoted as $\:{F}_{n}$​, is calculated as the product of the normal stiffness $\:{K}_{n}$​ and the overlap $\:{\delta\:}_{n}$​ in the normal direction. This relationship is given by Eq. (6):

6 $$\:{F}_{n}={K}_{n}{\delta\:}_{n}$$

The overlap parameter $\:{\delta\:}_{n}$​ represents the compressive displacement between contacting particles and is typically constrained to remain below 5% of the average particle radius to maintain model accuracy and stability, as supported by prior studies [10]. The contact stiffness values in the normal and shear directions, denoted as $\:{K}_{n}$​ and $\:{K}_{s}$​ respectively, are computed based on the intrinsic mechanical properties of the ballast’s parent rock. Specifically, these include the shear modulus $\:{G}_{s}$​ and Poisson’s ratio $\:\nu\:$. Drawing from the uniaxial compression experiments performed by Khatibi et al.^29^, which adhered to ISRM (1979)-EUR4 protocols, cylindrical rock cores with a 54 mm diameter and a 2.5 height-to-diameter ratio were tested. The findings indicated a Poisson’s ratio of 0.2 and a shear modulus of $\:{G}_{s}=8.9\:G$ for the ballast material, and corresponding stiffness values for different particle size distributions are summarised in Table 2, illustrating how contact parameters were tailored for the simulation.

**Table 2:** Mechanical and geometric parameters for contact force calculation based on particle size distribution. For comparison, the DEM simulation settings used in this study for real-shaped ballast were contrasted with both experimental benchmarks^29^and earlier DEM models employing spherical particles, as presented by Chalabii et al.^10^. The complete set of mechanical and contact parameters is summarised in Table 3.

PSD	Particles’ diameter (mm)	$\:{R}_{A}$ (mm)	$\:{R}_{B}$ (mm)	$\:{R}_{e}$ (mm)	$\:{\stackrel{\sim}{R}}_{e}$ (mm)	$\:{\delta\:}_{n}\:\left(mm\right)$	$\:{K}_{n}\:$(Pa)	$\:{K}_{s}\:$(Pa)
No.24	63.4, 38.1, 19	31.7	19.05	23.8	17	0.85	$\:0.4\times\:1{0}^{8}$	$\:0.53\times\:1{0}^{8}$
		9.5	19.05	12.7				
		31.7	9.5	14.6				
Samples 1, 2, 3 and 4	38.1, 19	19.05	9.5	12.7	12.7	0.635	$\:0.3\times\:1{0}^{8}$	$\:0.36\times\:1{0}^{8}$

**Table 3:** Mechanical and contact parameters used in DEM simulations for real-shaped ballast, compared with experimental values from Khatibi et al.^29^ and DEM simulations with spherical particles from Chalabii et al.^10^.

Parameters of Contact	Symbol	Unit	Value in Simulation for real shaped ballast	Experimental Value^29^	Value in Simulation for spherical particles^10^
Shear elastic modulus	$\:G$	$\:Pa$	–	$\:8.9\times\:{10}^{9}$	–
Poisson’s ratio of ballast	$\:\nu\:$	–	–	$\:0.2$	–
Ballast particle density	$\:{\gamma\:}_{b}$	$\:Kg/{m}^{3}$	$\:2600$	$\:2600$	$\:2600$
Sleeper clump density	$\:{\gamma\:}_{s}$	$\:Kg/{m}^{3}$	$\:2500$	$\:2500$	$\:2500$
Coefficient of inter-particle friction	$\:FI$	–	$\:0.9$	$\:0.9$	$\:0.9$
Side wall friction coefficient	$\:{f}_{ws}$	–	$\:1\times\:1{0}^{8}$	–	$\:1\times\:1{0}^{8}$
Base wall friction coefficient (subgrade)	$\:{f}_{wb}$	–	$\:0.9$	–	$\:0.9$
Wall normal and shear stiffness	$\:{K}_{sw},\:{K}_{nw}$	$\:Pa$	$\:0.57$	$\:0.57$	$\:0.57$

### Geometry of the model

**Fig. 8:**
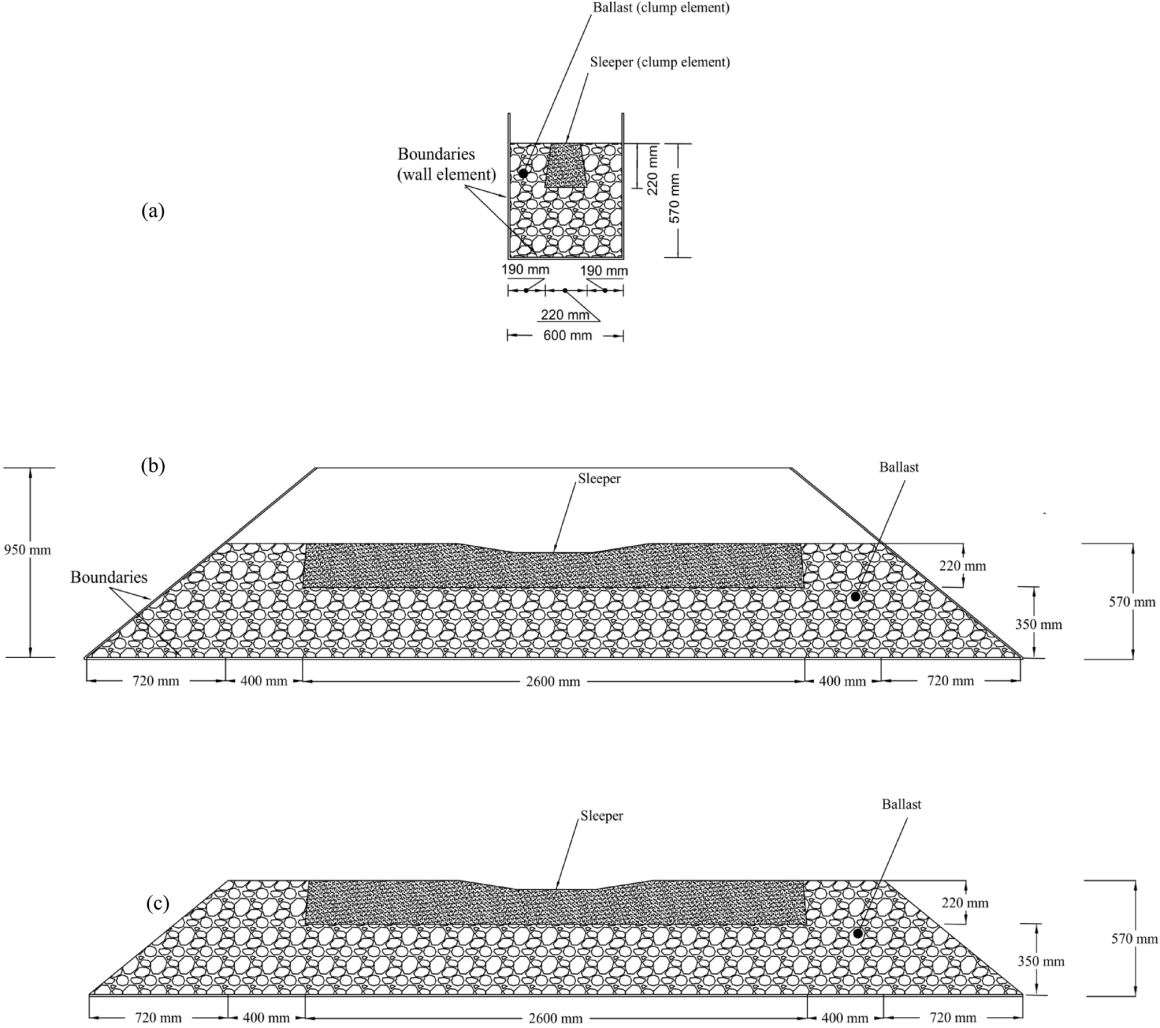
Illustration of the simulation setup: (a) longitudinal profile of the full model, (b) transverse section after removing walls aligned with the direction of applied lateral load, and (c) transverse section showing the model with lateral confinement walls intact.

A schematic representation of the developed model is provided in Fig. 8. The geometry consists of a trapezoidal prism designed to simulate a segment of the railway track structure. The model spans 4.84 m along its base, tapers to 1.95 m at the top, and stands 1 m in height, with a constant width of 0.6 m across its section. The selected base length of 4.84 m allows for the accurate representation of a track segment, including a ballast shoulder approximately 40 cm wide on each side of the sleeper. The domain height was chosen to ensure the accommodation of a sufficient volume of ballast particles without overlapping during the generation phase, thus achieving a realistic ballast packing for simulation purposes.

Following the gravitational deposition of ballast particles and the subsequent placement of the sleeper, surplus particles were carefully removed to achieve a ballast layer height of approximately 570 mm, as illustrated in Fig. 8b. Once the ballast surface was shaped to reflect field conditions, the side walls oriented along the intended lateral loading direction were eliminated to enable realistic deformation responses. The model width was fixed at 600 mm as shown in Fig. 8a, corresponding to the standard spacing requirements for concrete sleepers. To simulate boundary effects, rigid walls were employed along the model’s bottom and remaining sides; however, recognizing that these rigid boundaries do not perfectly mimic natural ballast-subgrade interactions, a realistic friction coefficient was assigned for contacts between ballast particles and between ballast and the underlying surface. The overall track section and its dimensions are clearly defined through the schematic presented in Fig. 8c.

### Simulation of ballast particles

The discrete element modeling (DEM) of ballast particles was carried out using PFC3D 7.0, with a primary focus on accurately replicating particle geometry and gradation effects. A total of five ballast samples with varying particle size distributions (PSDs) were modeled. Among these, Sample 5 corresponds to the No. 24 PSD, which was previously utilized by Khatibi et al. in experimental studies and Chalabii et al. in numerical simulation^10,29,37^. This particular gradation was selected to enable validation of the numerical model by comparing DEM-simulated lateral resistance results with laboratory test data from that study. To replicate the real morphology of the ballast particles, each sample was scanned using a high-resolution Artec Space Spider 3D scanner, and the scanned STL files were imported into PFC3D. These shapes were converted into clumps by densely filling the scanned geometry with spherical elements, thus preserving the particle’s angular features while enabling efficient DEM simulation. The resulting clumps reflect realistic contact behavior, including interlocking and surface friction, which are highly influenced by angularity. Following model validation using Sample 5, the remaining samples, each with distinct gradations and angularity levels, were simulated to examine how particle shape and distribution affect the lateral resistance of concrete sleepers.

### Sleeper’s simulation

**Fig. 9:**
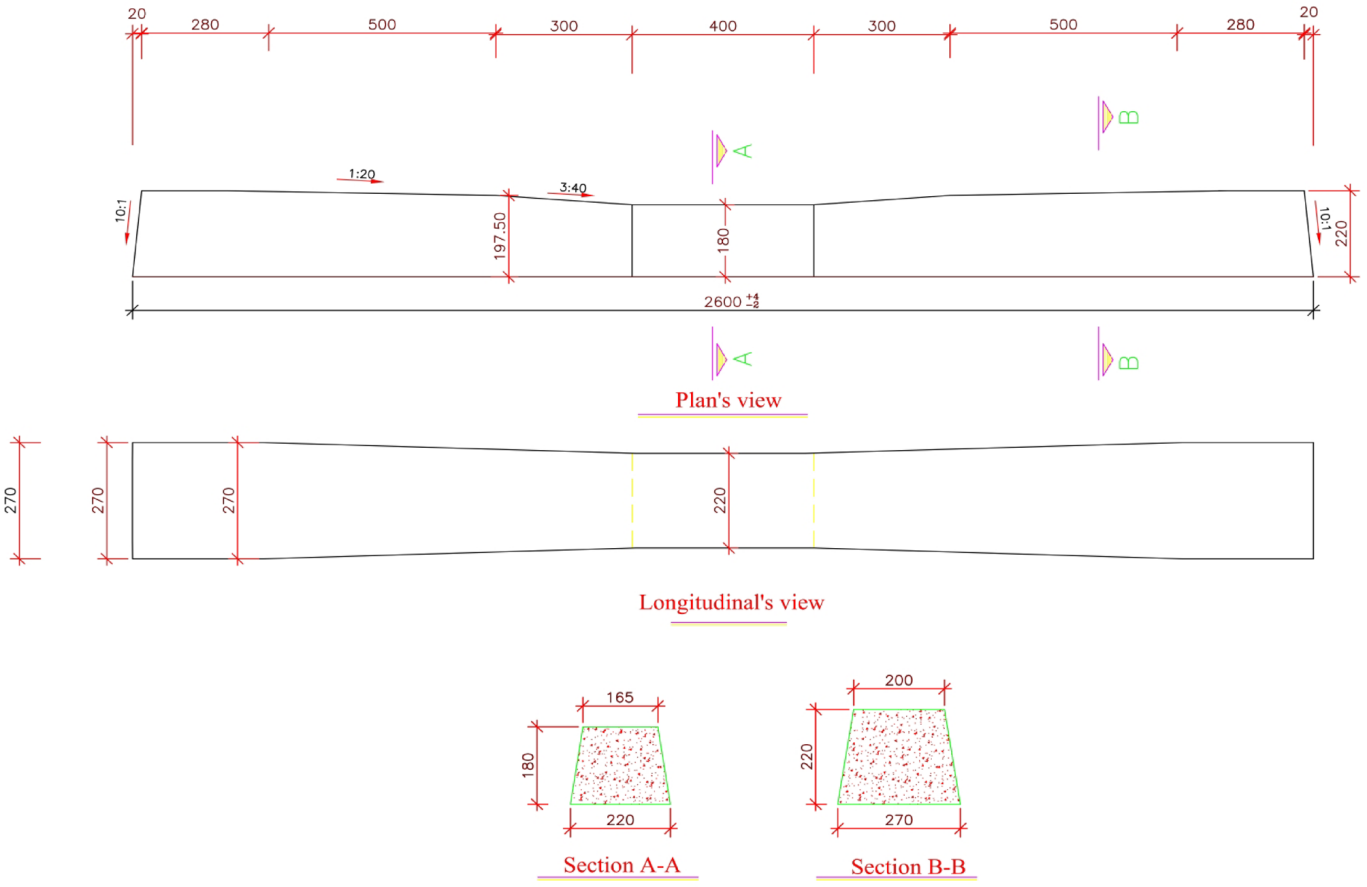
Dimensions of the B70 concrete sleeper used in the simulation, shown in millimetres.

In this study, the sleeper was modeled as a rigid clump in PFC3D, replicating the geometry of a standard B70 prestressed concrete sleeper, which is widely employed in European rail infrastructure^24^. The sleeper’s mass, dimensions, and positioning were selected to align with real-world design specifications, ensuring realistic interaction with the ballast. The detailed geometry of the sleeper used in the simulation is illustrated in Fig. 9.

**Fig. 10:**
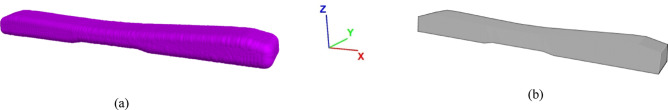
Representation of the concrete sleeper: (a) sleeper constructed using clump particles in PFC3D, and (b) 3D model of the B70 sleeper exported as an STL file.

To construct the sleeper in the numerical model, overlapping spheres were arranged to approximate the shape of the B70 sleeper. The clumped structure was generated from an STL-based geometry, which preserved the main contact features while maintaining computational tractability. Figure 10 shows the imported STL file alongside the generated clump structure used in the simulation. The sleeper was treated as a non-deformable rigid body to isolate the influence of ballast angularity on lateral resistance and was centrally positioned within the ballast bed.

### Simulation of whole model

**Fig. 11:**
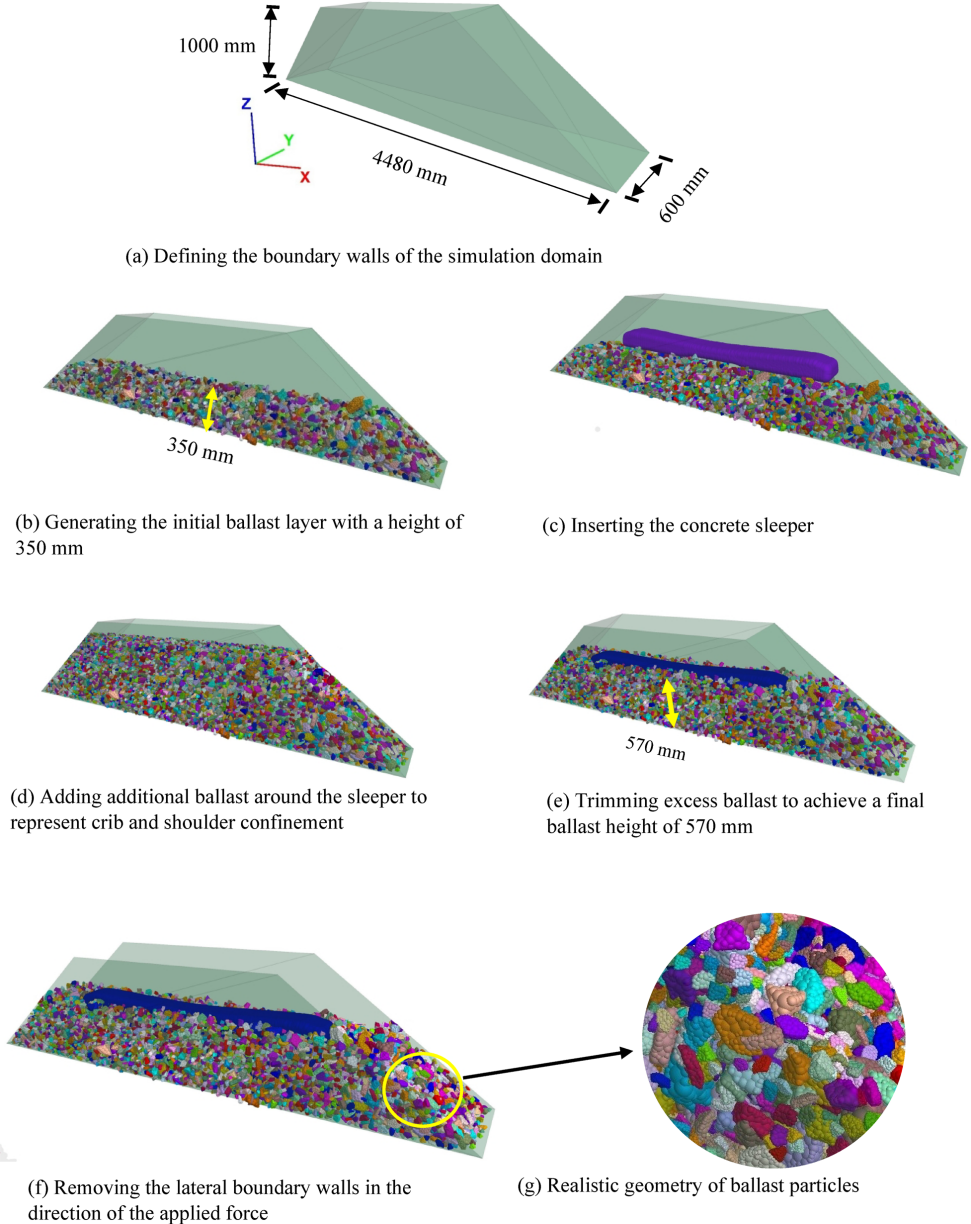
Step-by-step development of the simulation model.

**Fig. 12:**
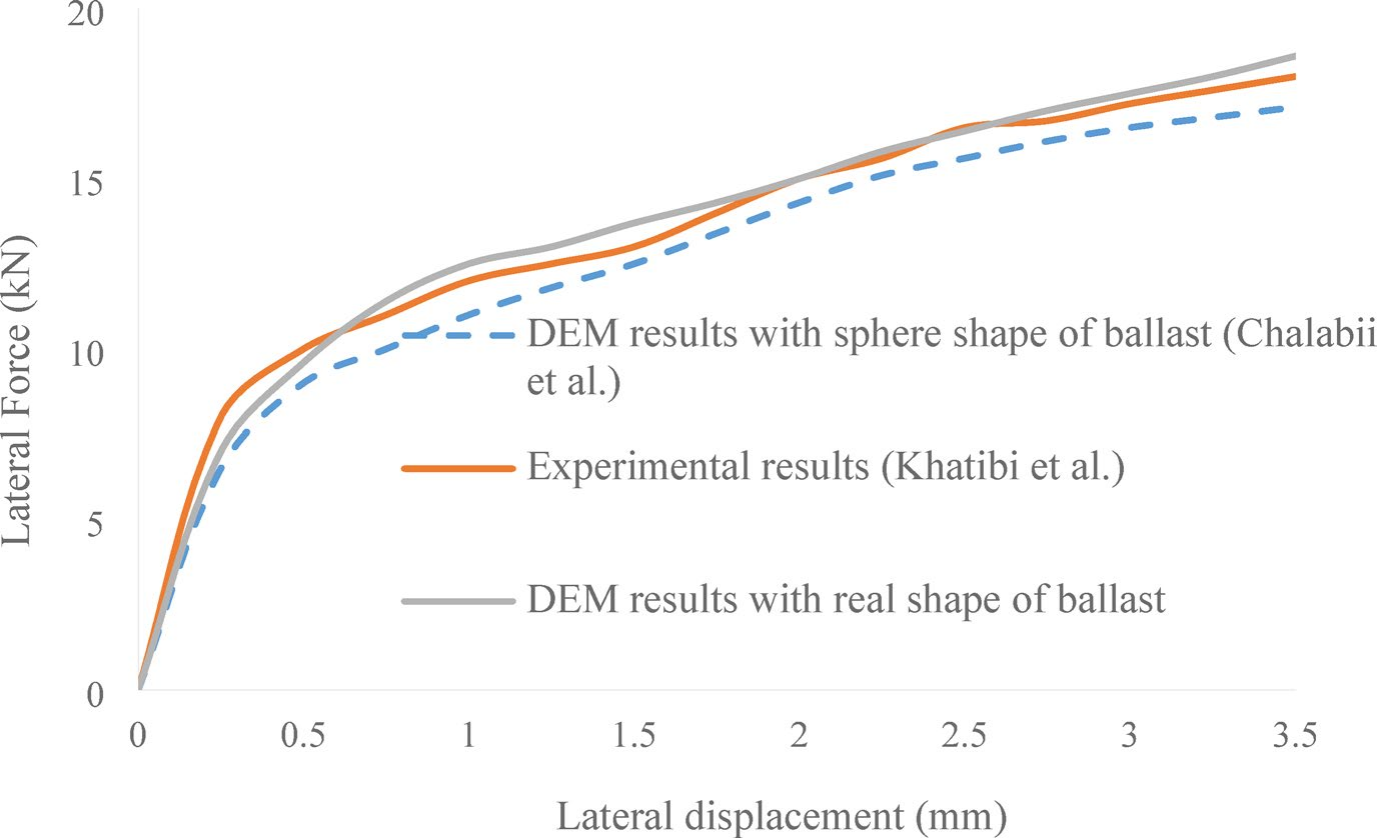
Validation of DEM simulation results using the real 3D-scanned shape of ballast particles, compared with experimental data^29^ and DEM results based on spherical ballast geometry^10^.

The ballast–sleeper interaction model was developed through a series of methodical steps using the PFC3D platform. The process began with the definition of the simulation domain, where base and lateral boundary walls were introduced to establish physical constraints as shown in Fig. 11a. These walls were assigned suitable contact properties to realistically emulate field-like confinement during loading. Subsequently, ballast particles were generated in accordance with the particle size distributions specified in Fig. 12. The geometrical shape of each particle was constructed using STL-imported clumps, previously derived from 3D-scanned samples. Gravity loading was applied to allow the ballast particles to naturally settle and compact. To account for variations in final layer height and ensure a dense packing, excess particles were initially introduced. After stabilization, surplus ballast was removed, forming a compacted layer approximately 350 mm thick to represent the ballast bed which can be seen in Fig. 11b. Following the ballast preparation, the sleeper modeled as a rigid clump that refer to Fig. 11c, was inserted into the center of the bed and allowed to settle under its own weight until vertical equilibrium was achieved. As shown in Fig. 11 d, the crib and shoulder regions surrounding the sleeper were then filled with additional ballast particles. This was followed by another trimming stage to remove surplus material and define a final model height of 570 mm, representing the full ballast cross-section that is illustrated in Fig. 11e. To replicate realistic boundary conditions during lateral loading, the side walls in the direction of applied force were removed as shown in Fig. 11f. This allowed the sleeper to move freely during loading, thereby simulating actual in-track behavior under lateral loads. The entire assembly was then subjected to a Single Tie Push Test (STPT), wherein lateral displacement was incrementally applied at the sleeper’s center. In addition, the real shape of modelled ballasts is presented in Fig. 11g. The simulation progressed under a quasi-static loading regime, ensuring gradual force application and accurate evolution of particle contacts. This structured simulation workflow facilitated the precise examination of lateral resistance as influenced by ballast particle angularity, contributing valuable insights into sleeper stability in ballasted track systems.

## Results

### Model validation and verification

Figure 12presents a comparison of lateral resistance behavior obtained through experimental testing by Khatibi et al.^29^, DEM simulation using idealised spherical ballast particles as modeled by Chalabii et al.^10^, and the current study’s DEM simulation incorporates the real 3D-scanned shapes of ballast particles. The horizontal axis shows lateral displacement, while the vertical axis represents lateral force ($\:kN$). As illustrated, the simulation results using real ballast geometries display a close agreement with the experimental data across the entire displacement range. This alignment is particularly notable in the early stages of loading, where the initial stiffness and rapid increase in lateral force are captured more accurately. In contrast, simulations using spherical particles consistently underestimate lateral resistance, demonstrating a smoother and less steep response curve. This improvement indicates how realistic angular geometries more accurately replicate load-transfer and interlocking mechanisms. It should be mentioned, though, that the model utilizes various particle size distributions (PSDs) to represent degradation rather than directly simulating grain fracture, as it assumes rigid particle clumps.

Adopting a rigid confining mold (inner diameter = 203 mm, in accordance with ASTM D 3398^38^) and calibrating the wall–particle friction coefficient ($\:{K}_{sw}=\:{K}_{nw}=0.57$) to match experimental behavior reduced boundary effects. There were no signs of large particles being artificially contained close to the walls.

The adopted DEM approach provides a dependable representation of ballast lateral behavior under controlled conditions, as confirmed by the close agreement between experimental and numerical curves. The improved correlation between the DEM results and experimental outcomes confirms the effectiveness and reliability of the modeling approach adopted in this study. The validation procedure has been strengthened. The DEM results were compared with laboratory data by Khatibi et al.^39^, reproducing the experimental lateral force–displacement curve within 5%deviation. The unbalanced force ratio reached less than 1 × 10⁻⁴, satisfying standard DEM equilibrium criteria. These validation details confirm that the simulated ballast system achieved mechanical stability consistent with accepted DEM modeling practices.

### Quantification of ballast degradation

**Fig. 13:**
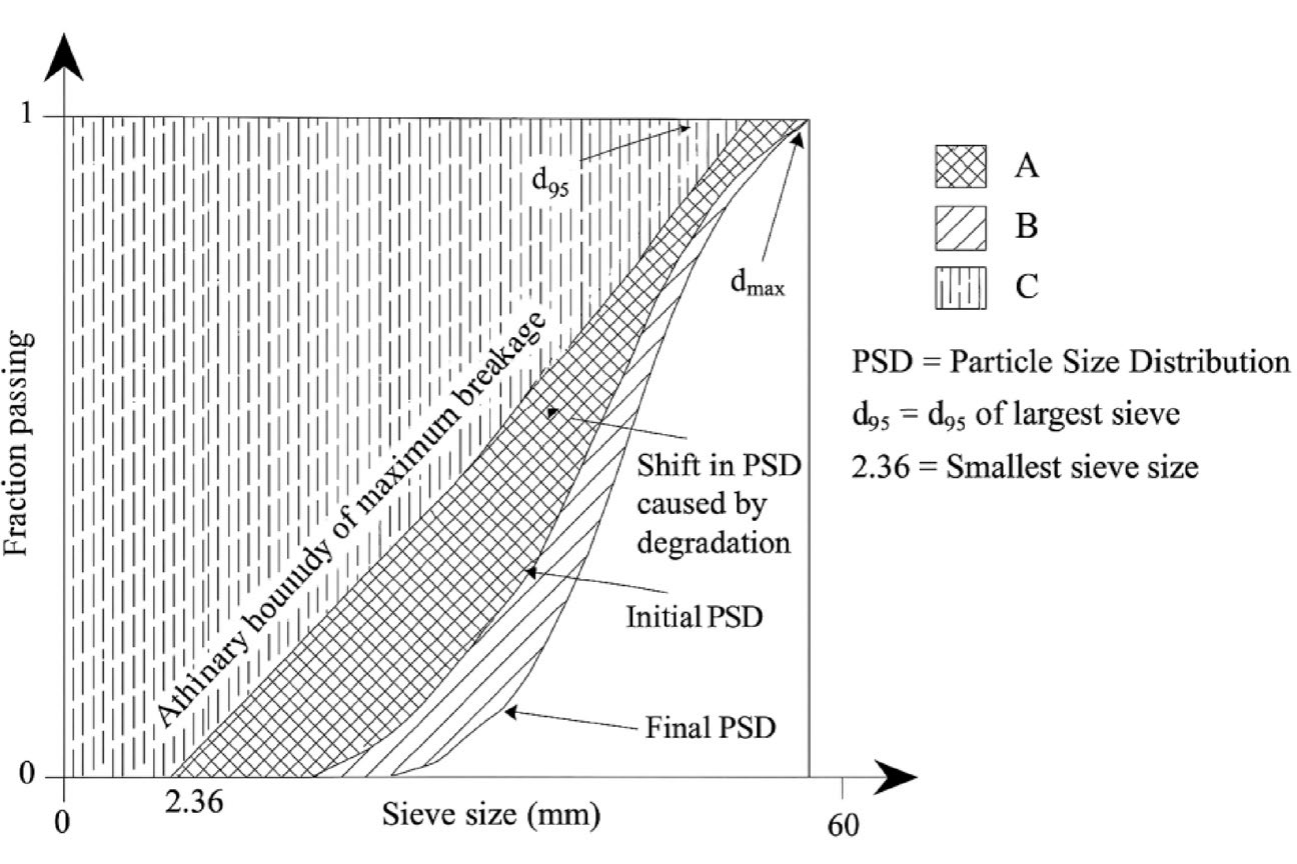
Evaluation of the Ballast Breakage Index ($\:BBI$), as modified according to Indraratna et al.^38^, and the Breakage Ratio ($\:{B}_{r}$), determined based on the approach outlined by Marsal^40^.

In this research, the quantification of ballast degradation and its impact on lateral resistance was assessed through two primary indicators: $\:BBI$ and $\:{B}_{r}$. The $\:BBI$ was computed based on a method adapted from Hardin’s approach and further refined by Indraratna et al.^38^, defined by Eq. (7) as the ratio of area $\:A$ to the sum of areas $\:A$ and $\:B$:

7 $$\:BBI=\frac{A}{A+B}$$

In this context, area $\:A$ represents the region enclosed between the initial and final particle size distribution curves, while area $\:B$ extends from the final PSD to a defined limiting boundary, quantifying the degree of particle fragmentation. To further characterize ballast degradation, the Breakage Ratio ($\:{B}_{r}$) was also introduced^40^, offering complementary insights into the degradation process. $\:{B}_{r}$ is defined by Eq. (8) as the ratio of potential breakage $\:{B}_{p}$ to total breakage $\:{B}_{t}$:

8 $$\:{B}_{r}=\frac{{B}_{p}}{{B}_{t}}$$

In this formulation, $\:{B}_{p}$ is equivalent to area $\:A$, and $\:{B}_{t}$, representing the total breakage, is calculated as the sum of areas $\:C$ and $\:A$, as shown in Eq. (9):

9 $$\:{B}_{t}=C+A$$

A schematic of these areas ($\:A,\:B,\:C$) is presented in Fig. 13. Because DEM clumps are rigid, these indices are not derived from simulated particle fracture but rather from PSD differences before and after numerical testing, consistent with Indraratna et al.^38^. This approach captures the effect of degradation, not the fracture mechanism itself.

### Limitations and interpretation of PSD-Based breakage representation

In the DEM environment, ballast particles were simulated in this study as inflexible, non-breakable clumps. As a result, no explicit fracture or fragmentation processes were modeled. Rather, various preset particle size distributions were used to indirectly depict the impact of degradation. Assuming that breakage and abrasion had taken place before testing, each PSD (Samples 1–5) was made to represent a unique pre-degradation state. Therefore, rather than using DEM-induced changes during simulation, the $\:BBI$ and $\:{B}_{r}\:$were calculated from the differences among these initial PSDs. Computational efficiency and controlled comparison between gradations with different assumed degradation levels are provided by this indirect approach. But unlike bonded-particle or hybrid breakage models, it does not account for the mechanics of particle fracture, asperity wear, or contact evolution. Therefore, in this context, BBI and Br are not direct measures of breakage within the DEM, but rather proxies for degradation level. More realistic assessment of ballast fragmentation mechanisms would be possible in the future if bonded-particle models were used to explicitly simulate crack initiation and propagation.

### Lateral resistance evaluation and correlation analysis

**Fig. 14:**
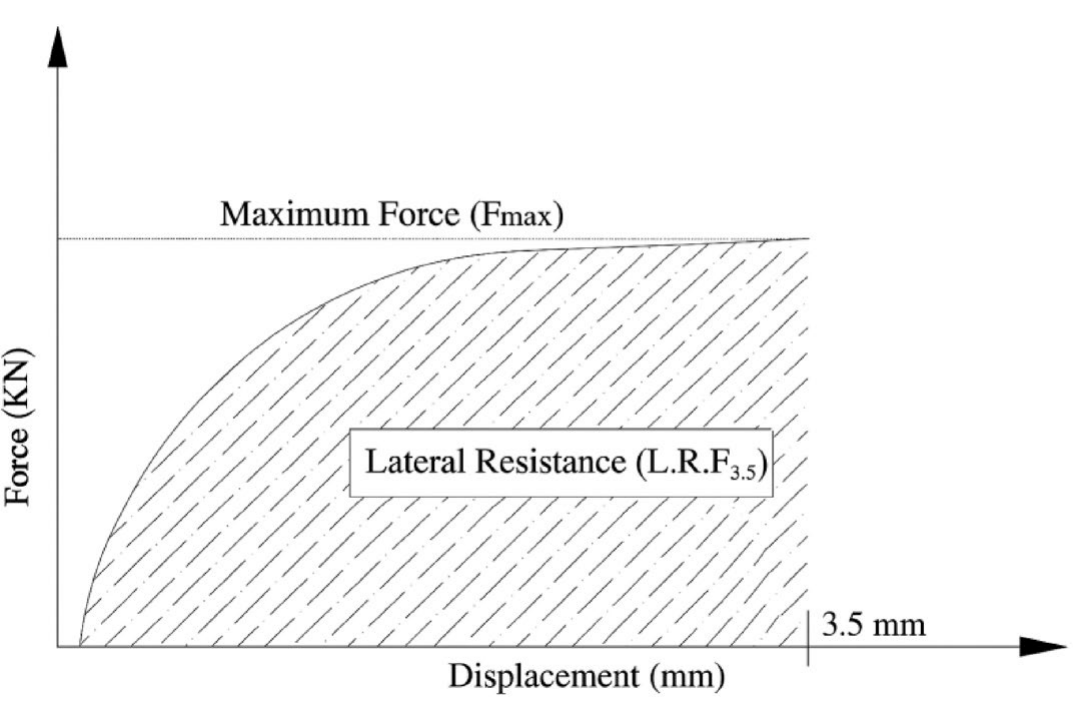
Definition of the lateral resistance factor ($\:{LRF}_{3.5}$), representing the area under the lateral force–displacement curve up to 3.5 mm of displacement, as proposed by Chalabii et al.^37^.

To quantify the lateral resistance of concrete sleepers, Chalabii et al.^37^ introduced the Lateral Resistance Factor ($\:{LRF}_{3.5})$ as the area under the force–displacement curve up to 3.5 $\:mm$ of displacement, which expressed by Eq. (10):

10 $$\:{LRF}_{3.5}={\int\:}_{0}^{3.5}{F}_{L}d\delta\:$$

where $\:{F}_{L}$ is the applied lateral force ($\:kN$) and $\:\delta\:\:$is the corresponding displacement ($\:mm$). A schematic illustration of this definition is presented in Fig. 14, showing how the lateral resistance factor ($\:{LRF}_{3.5}$) corresponds to the shaded area under the curve.

**Fig. 15:**
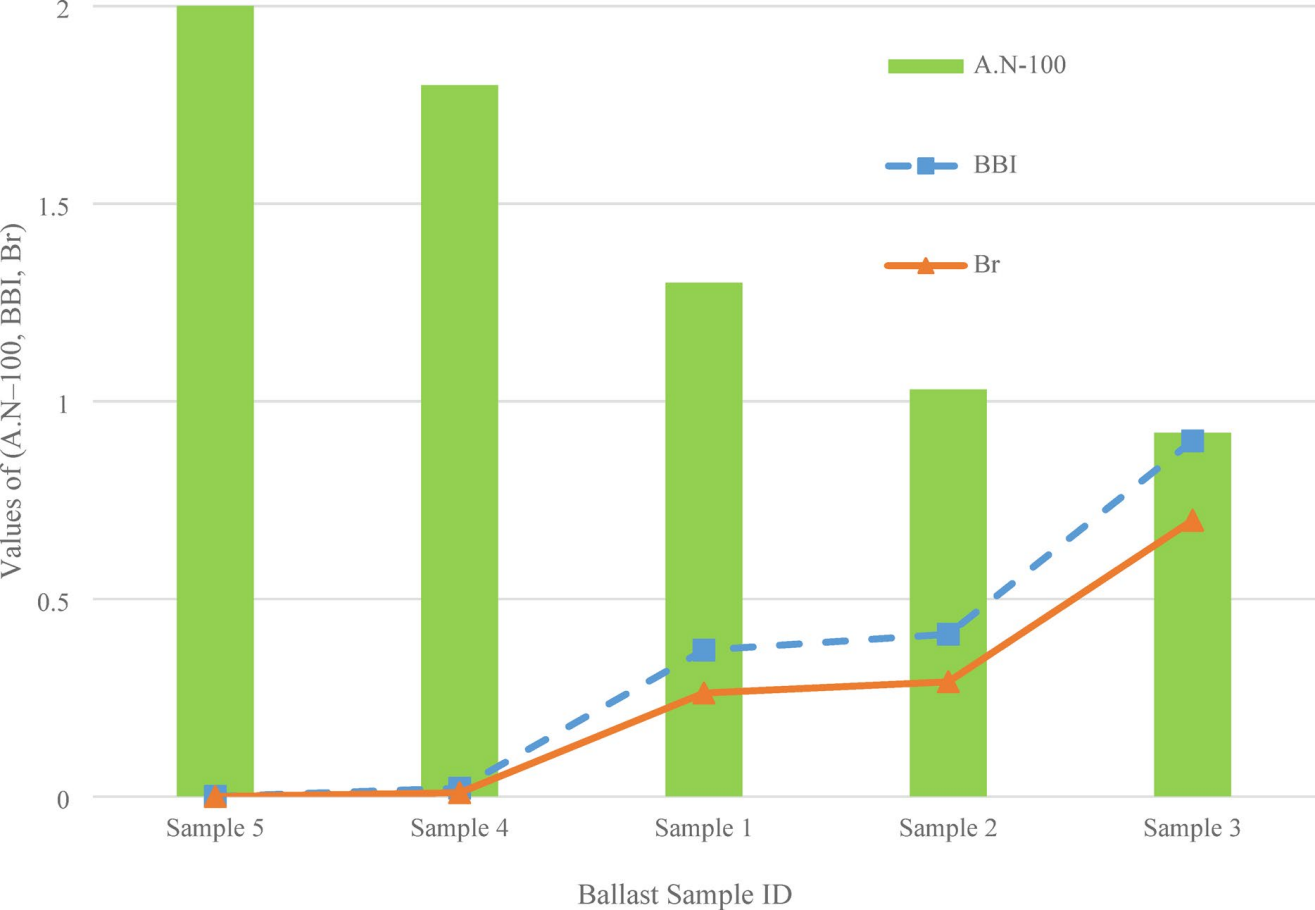
Comparative analysis of ballast samples with varying particle size distributions, illustrating the relationship among the adjusted Angularity Number ($\:AN\:\--\:100$), Ballast Breakage Index ($\:BBI$), and Breakage Ratio ($\:{B}_{r}$).

The chart presented in Fig. 15 illustrates the comparative behavior of ballast samples with different particle size distributions by plotting three parameters: to facilitate comparison between angularity and degradation indices, the Angularity Number was adjusted by subtracting 100 (i.e., $\:AN -100$). This linear transformation does not alter the relative trend but brings the numerical scale of AN closer to that of the Ballast Breakage Index ($\:BBI$) and Breakage Ratio ($\:{B}_{r}$), both of which vary between 0 and 1. This approach allows direct visualization of their inter-relationships without affecting physical interpretation. As shown, Sample 5, which adheres to PSD No. 24, exhibits the highest angularity 103 with no breakage with $\:BBI\:=\:0$, $\:{B}_{r}=0$. In contrast, Sample 3, which has the lowest angularity ($\:AN\:=\:101.92$), indicates the highest levels of degradation, with a $\:BBI$ of 0.901 and $\:{B}_{r}$ of 0.699. This inverse relationship between angularity and degradation is evident across the dataset: as the angularity number decreases, both the $\:BBI$ and $\:{B}_{r}\:$values increase. These findings indicate that more angular particles resist breakage more effectively, likely due to their enhanced interlocking behavior and greater surface roughness. This observation supports the hypothesis that ballast angularity plays a crucial role in maintaining structural integrity under lateral loads, consistent with prior research findings such as those by Krengel et al.^40^ and Chalabii and Movahedi Rad^10^.

**Fig. 16:**
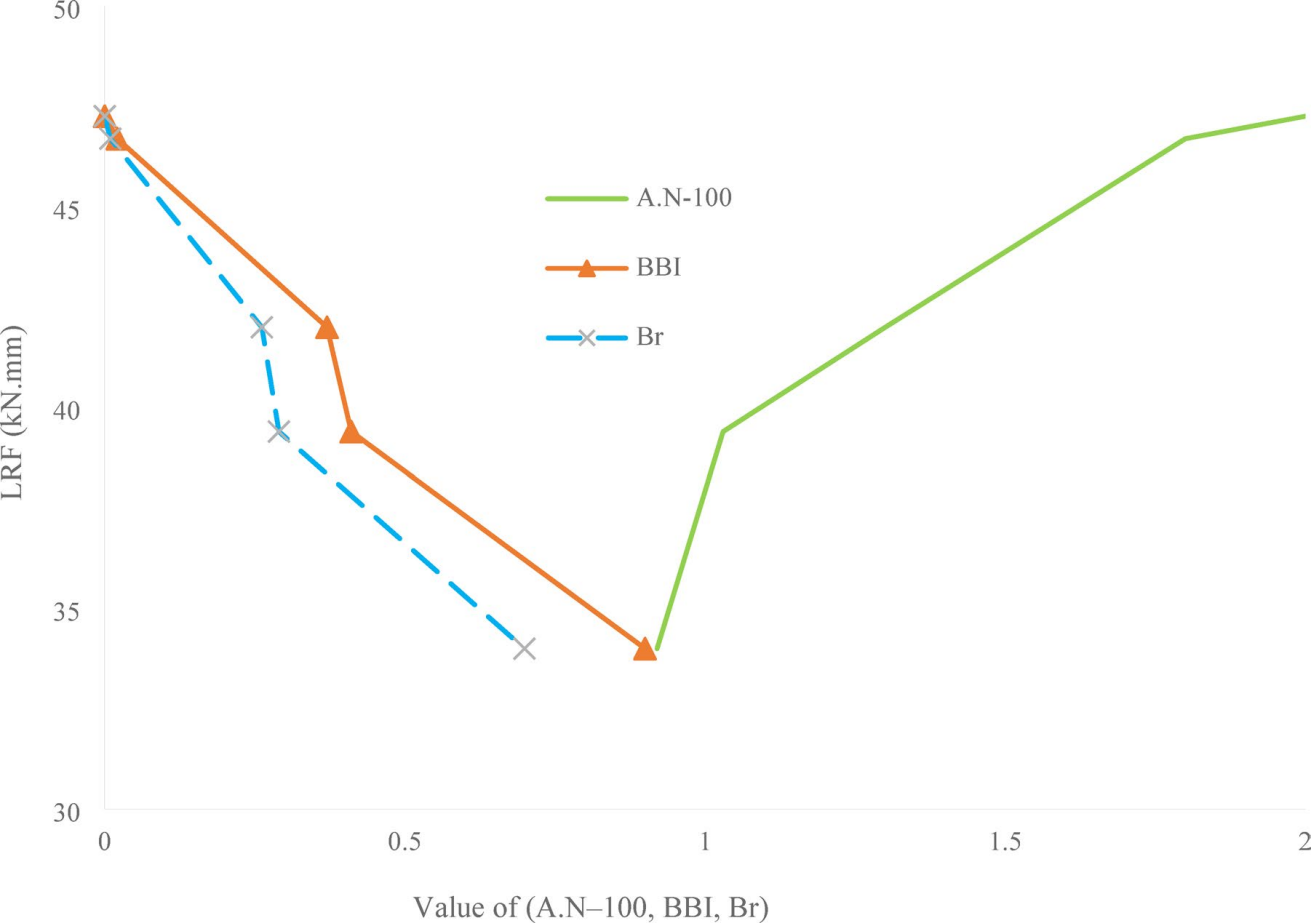
Correlation between $\:{LRF}_{3.5}$ and three normalized ballast characteristics: adjusted Angularity Number ($\:AN-100$), Ballast Breakage Index ($\:BBI$), and Breakage Ratio ($\:{B}_{r}$).

Figure 16 illustrates the relationship between $\:{LRF}_{3.5}$, measured in kN·mm, and three normalized ballast properties: the adjusted Angularity Number ($\:AN-100$),$\:\:BBI$ and $\:{B}_{r}$. On the x-axis, the values of $\:AN$, $\:BBI$, and Br have been normalized for better comparison, while the y-axis presents the corresponding $\:{LRF}_{3.5}\:$values obtained from DEM simulations for each ballast sample. The plot reveals a clear inverse relationship between both $\:BBI$ and $\:{B}_{r}\:$with $\:{LRF}_{3.5}$. As the values of $\:BBI$ and $\:{B}_{r}\:$increase, indicating higher levels of ballast degradation, the lateral resistance systematically decreases. This suggests that ballast breakage significantly weakens the interlocking behavior among particles, leading to lower shear strength and reduced resistance against lateral sleeper movement. For example, the highest values of $\:BBI$ and $\:{B}_{r}\:$are associated with the lowest $\:{LRF}_{3.5}\:$of approximately 34 kN·mm, highlighting the detrimental impact of particle degradation on track stability. Conversely, the $\:AN-100$ curve shows a positive correlation with $\:{LRF}_{3.5}$. As the adjusted angularity increases, $\:{LRF}_{3.5}\:$also rises, suggesting that more angular particles enhance interlock and frictional contact, thereby improving resistance against lateral forces. The highest $\:{LRF}_{3.5}$ value corresponds to the greatest angularity, indicating the importance of particle shape in mechanical performance. This combined plot underscores that angularity positively influences lateral resistance, while ballast degradation reflected in higher $\:BBI$ and $\:{B}_{r}$ has a negative effect. Together, these trends emphasize the critical role of maintaining angularity and minimizing degradation to preserve ballast effectiveness and ensure track stability.

Although the current DEM results quantitatively show how angularity affects lateral resistance, field-scale validation is necessary before they can be directly applied in railway engineering. However, the known relationships between lateral strength and angularity can guide maintenance planning and ballast selection, maximizing materials for better track stability over the long run.

## Conclusion

This research used a combination of laboratory testing, 3D geometry capture, and discrete-element modeling (DEM) to examine the effects of ballast particle angularity on the lateral resistance of concrete sleepers. Five ballast samples with varying particle size distributions (PSDs) had their angularity numbers ($\:AN$) measured in order to quantify changes in surface roughness and particle shape. The DEM model was verified against laboratory data using Sample 5, which complies with standard PSD No. 24. Ballast fragments were digitized using an Artec Space Spider 3D scanner to create realistic particle morphology, and the resulting STL files were then transformed into clumps for PFC3D simulation. This helped the numerical model better represent interlocking behavior under lateral loading while maintaining the angular characteristics of the particles. It was not possible to directly model the physical process of particle breakage because PFC3D clumps are inflexible and do not fracture. Assuming that breakage and abrasion had already taken place before testing, various PSDs were used to indirectly depict the effects of degradation; this method is in line with recent research like that done by Chalabii et al. (2024).

The main findings can be summarized as follows:

- Validation: Compared to models based on spherical particles, DEM simulations employing real-shaped clumps more faithfully captured the experimental force–displacement response.
- Impact of angularity: Angularity and lateral resistance were found to be strongly positively correlated. Greater load-carrying capacity and better interlocking were indicated by more angular ballast.
- Degradation Effect: Ballast Breakage Index ($\:BBI$) and Breakage Ratio ($\:Br$) increased in samples with lower angularity (and higher degradation level), suggesting a loss of stability.
- Implication for practice: Sleeper lateral resistance can be increased and track service life extended by maintaining angular particle geometry through proper material selection and handling.
- Large-scale field validation and repeated simulations under various boundary conditions were not included in this study because it was primarily concerned with laboratory-scale modeling; these topics will be covered in subsequent studies.
- Limitation: Individual grain fracture and asperity wear are not captured by the PSD-based degradation model that was adopted; future research will expand the model by explicitly simulating breakage using bonded-particle or particle-replacement techniques. Also, the present study employed one representative DEM simulation per gradation condition. Future research will expand to parametric studies under varying boundary conditions to improve statistical confidence.

Overall, the findings indicate how important particle angularity is to the mechanical performance of ballasted tracks and offer a verified framework for integrating sleeper lateral resistance analysis with real-shape ballast modeling.

## Data Availability

All data generated or analysed during this study are included in this published article.

## List of symbols

PSD: Particle Size Distributions
DEM: Discrete Element Method
STPT: Single Tie Push Test
$\:BBI$: Ballast Breakage Index
$$\:Br$$: Breakage Ratio
USP: Under-sleeper pads
CR: Crumb Rubber
PSB: Polyurethane-Solidified Ballast
CWR: Continuously Welded Rail
ASTM: American Society for Testing and Materials
LED: Light-Emitting Diode
STL: Standard Triangle Language
UDEC: Universal Distinct Element Code
3DEC: Three-Dimensional Distinct Element Code
PFC: Particle Flow Code
$$\:AN$$: Angularity numbers
$\:{LRF}_{3.5}$: Lateral resistance force for 3.5 mm displacement ($\:kN.mm$)
$\:{V}_{c}\left(\%\right)$: Uncompacted void content
$\:{V}_{m}$: Volume of the mold ($\:{cm}^{3})$
$\:{M}_{b}$: The mass of the ballast sample ($\:g)$
$\:{G}_{b}$: Specific gravity of the aggregate
$\:{\beta\:}_{w}$: The density of water ($\:g/{cm}^{3})$
$\:{F}_{n}^{l}$: Normal force components ($\:N)$
$\:{F}_{s}^{l}$: tangential force components ($\:N)$
$\:{F}_{n}^{d}$: Damping forces in the normal direction ($\:N)$
$\:{F}_{s}^{d}$: Damping forces in the shear direction ($\:N)$
$\:{\beta\:}_{n}$: Damping ratios in normal direction
$\:{\beta\:}_{s}$: Damping ratios in tangential direction
$$\:{g}_{s}$$: A surface gap ($$\:m)$$
$\:\mu\:$: The coefficient of friction
$\:{M}_{l}$: Linear force computation mode
$$\:{M}_{d}$$: Damping mechanism
$\:{F}_{c}$: Contact force vector ($\:N)$
$\:M$: The resulting contact moment
$\:{K}_{n}$: ​The normal stiffness values ($\:N/m)$
$\:{K}_{s}$: The tangential stiffness values ($\:N/m)$
$\:{G}_{s}$: The shear modulus ($\:Pa)$
$$\:\nu\:$$: Poisson’s ratio
$\:{R}_{A}$: Radius of particle A ($\:m)$
$\:{R}_{B}$: Radius of particle B ($\:m)$
$\:{R}_{e}$: The effective particle radius ($\:m)$
$\:{F}_{n}$: Normal contact force ($\:N)$
$\:{\delta\:}_{n}$: ​The compressive displacement between contacting particles ($\:m)$
$$\:{B}_{p}$$: Potential breakage ($$\:m)$$
$$\:{B}_{t}$$: Total breakage ($$\:m$$)
